# Using Carrots Not Sticks to Cultivate a Culture of Safeguarding in Sport

**DOI:** 10.3389/fspor.2021.625410

**Published:** 2021-03-02

**Authors:** Judith L. Komaki, Yetsa A. Tuakli-Wosornu

**Affiliations:** ^1^Department of Psychology, Baruch College, City University of New York, New York, NY, United States; ^2^Department of Chronic Disease Epidemiology, School of Public Health, Yale University, New Haven, CT, United States

**Keywords:** reinforcement, reward, organizational culture, climate, safety management, sexual harassment, emotional abuse, sports

The power-driven, win-at-all-costs milieu of many sport settings can create fertile ground for athlete victimization and abuse (Roberts et al., 2020). Victory can in fact be so sovereign that abusive coaches and staff are enabled and “even rewarded…in the name of winning” (Armour, 2020). Athlete abuse prevention therefore requires systemic cultural change (Letourneau et al., 2014; Rhind and Owusu-Sekyere, 2017). Thus far, however, enacting this idea has eluded organizations in sport (Mountjoy et al., 2016; Harris and Terry, 2019; Kerr et al., 2019; Rhind and Owusu-Sekyere, 2020) as well as in other settings (National Academies of Sciences, 2018; Fort Hood Independent Review Committee, 2020). Moreover, authority figures in sport^1^ have historically hindered abuse prevention efforts. As activist reformer Brackenridge (2001) wrote, their “collective denial effectively blinded [them] to the possibilities that they might actually be harboring or facilitating sexual [and others forms of] exploitation”.

This opinion piece first identifies the limitations facing current approaches to athlete abuse prevention. It then offers a novel solution: an athlete-centered safeguarding strategy based on positive reinforcement theory (Skinner, 1953). This approach, as described in Komaki and Minnich (2016), will enable sports authorities to transform the culture, the most powerful predictor of victimization and abuse (National Academies of Sciences, 2018).

## The Current Culture of (Elite) Sport Facilitates Abuse

Sport's cultural terrain, especially at the elite level, is challenging for athletes and sports authorities to navigate (Roberts et al., 2020). Former CEO of the U.S. Olympic and Paralympic Committee Scott Blackmun personified the predicament that places athletes in vulnerable positions. Whether wooing coaches, executives, or sponsors, Blackmun prioritized putting medals around athletes' necks over keeping them safe. “For us,” Blackmun said in 2014, “it's all about medals” (Hobson, 2018). This single-minded focus on winning in many sport settings is so entrenched that abusive behaviors are perceived to have beneficial effects. This false belief and others are major organizational drivers of all forms of abuse (Fortier et al., 2020; Roberts et al., 2020). As gymnastics coach Gerrit Beltman admitted, “it was never my conscious intention to beat (athletes), to yell at them, to hurt their feelings, to belittle them, to gag them… But it did happen… I went too far because I thought it was the only way to instill a winning mentality in them” (Macur, 2020a).

Despite evidence that good interpersonal health improves athletic performance (Al-Yaaribi et al., 2018; Cascagnette et al., 2020), many sports organizations still have difficulty prioritizing safety (Grey-Thompson, 2017; Kerr and Stirling, 2019) and explicitly connecting athlete welfare to winning. Recently, however, some sports authorities have displayed welcome attitude changes. The head of U.S.A. Gymnastics Li Li Leung, for example, contended in 2020 that well-being and victory are not mutually exclusive. “We believe that our athletes can be competitively excellent and compete at a very high level and also be happy and feel safe,” she said (Macur and Allentuck, 2020).

Although figures like Leung are well-intentioned, they lack the means to ensure athlete well-being because they know only when their organization has failed: when they get a report of abuse. If their only safeguarding metric consists of fumbles and failures, they cannot measure the opposite: how well their organization is doing in making athletes feel safe.

## Abuse Prevention Strategies That Rely Heavily on Reporting Have Limitations

Sports organizations currently depend almost exclusively on systems of disclosures and sanctions to deter abuse (Vertommen et al., 2013; Mountjoy et al., 2016; U.S. Center for SafeSport, 2021a). Only after a victim or observer takes the risk of complaining does the punishment-redress process commence. Thus, the onus for abuse prevention is often placed on the most vulnerable member of the sports system, the athlete. Alas, this over-reliance on reporting is unsubstantiated (Letourneau et al., 2014).

Reporting-initiated prevention efforts are further complicated by people's general reluctance to disclose abuse. In the case of sexual abuse, for example, only 10% of female victims ever file complaints; for male victims, only 5% (Stop Street Harassment, 2018). Even when athletes and others finally disclose harm, they often wait years, as Diana Nyad did before confiding in her best friend about her abusive high school coach (Nyad, 2017). Among the reasons so few report are: victims may be re- and further traumatized through reporting (van der Kolk, 2002; McClinton Appollis et al., 2015), and in sport, there are powerful cultural forces (“grin and bear it,” “no pain no gain” attitudes) and entrenched power imbalances (Roberts et al., 2020) that discourage disclosure of harms and *actively* undermine reporting programs.

Finally, sports authorities do not always take the necessary action following abuse reports. The belief that “no news is good news” fosters an atmosphere of stuffing reports in desk drawers (Kwiatkowski et al., 2016), dismissing victims' complaints (Allentuck, 2019), or even retaliating against those who report (Denhollander, 2018). In order to maintain their reputations, for example, Pennsylvania State University's football coach Joe Paterno and other top administrators “stood-by in silence or actively concealed knowledge of abuse” (Hartill, 2013). The failure to follow through persists at the highest levels: an 18-month investigation in 2019 found that two of the top-ranking U.S. Olympic and Paralympic Committee officials, Scott Blackmun and Alan Ashley, did little to probe, report, or halt Larry Nassar, the long-standing gymnastics team doctor and now convicted sexual predator (McPhee and Dowden, 2018). Olympian Aly Raisman noted ruefully how quick officials were to “capitalize on and celebrate my success. But did they reach out when I came forward (to report abuse)? No,” she said (Raisman, 2018).

## Crafting the Culture Using Carrots Not Sticks

Given the foregoing, simple fixes to athlete abuse prevention won't do. Nothing short of a cultural revolution is required. A safeguarding model in which teams are rewarded for cultivating a constructive culture—as judged by athletes—is an innovative approach in sport. Fostering a positive, athlete-centered culture demonstrates that sports organizations care and can also avert physical, sexual, and emotional abuse. Moreover, it empowers athletes' voices.

Undergirding this proposal is the theory of positive reinforcement (Skinner, 1953). This well-established theory focuses on the environment and provides a known driver of human motivation: favorable performance consequences. Thus, participants in sport may be *more* motivated to make meaningful behavior changes when positive consequences such as recognition and encouraging feedback (e.g., “carrots”) are used rather than negative consequences such as sanctions and dismissals (e.g., “sticks”).

The effectiveness of the reinforcement approach has been documented in rigorously controlled experiments outside of sport, with a success rate as high as 93% (Komaki et al., 2000). Reinforcement interventions have resulted in measurable behavioral improvements ranging from increased work productivity to better customer service in private and public sectors. The approach was used to prevent workplace accidents in a food manufacturing plant, for example (Komaki et al., 1978). In this setting, senior leaders faced similar barriers as sport administrators do: employees rarely reported injuries due to fear of retaliation, and leaders knew about employees' injuries only after serious accidents. After creating a metric for and recognition of safety performance, safety increased and accidents decreased (Komaki et al., 1978).

Using this carrots-rather-than-sticks model, the lead author (JLK) is now discussing a reinforcement-based abuse prevention plan with the U.S. Army. For this model to work, soldiers will be asked to respond to a “Trust Culture Checklist,” enabling feedback and recognition of soldiers and their supervisors.

## How Rewarding Teams and Entourages for Maintaining a Positive Culture Might Work in Sport

Sport administrators are invited to implement this model. For example, athletic directors could recruit school-based teams to create a positive culture using a “*Safeguarding Checklist*.” Modeled after the Army's “Trust Culture Checklist,” an example of a “Safeguarding Checklist” that could be used in sport is provided in Table 1. Athletes are asked to answer questions about the culture, which includes team camaraderie (Salas et al., 2020) and supportive leadership (Komaki, 1998), as well as their sense of personal well-being (Quick and Tetrick, 2011). If coaches and others engage in healthy athlete-centered behaviors, while teammates watch out for one another, the culture should improve and athletes' welfare should thrive.

**Table 1:** Safeguarding Checklist.

Think about your team over the past week and answer the following questions (check all that apply)		
**Camaraderie**		
In the last week, one or more **athletes** on my team would have or did…?		
1.Call or text me, e.g., about dinner, a movie	□ Yes	□ No
2.Ask me how I'm doing	□ Yes	□ No
3.Make sure I had transportation to grocery store, team events	□ Yes	□ No
4.Encourage me in my training, e.g., working harder, with more rest breaks	□ Yes	□ No
5.Celebrate with me when/if something good happens to me	□ Yes	□ No
6.Make me feel comfortable talking through a personal problem	□ Yes	□ No
7.Notice if/when something bad happens to me	□ Yes	□ No
8.Backed me if/when I had challenges with my team/coach/staff	□ Yes	□ No
9.Looked out for potentially unsafe situations	□ Yes	□ No
10.Intervened if/when someone bothered/pressured me	□ Yes	□ No
**Leadership**		
In the last week, my **coach** would have or did.?		
1.Check in with me/team about triumphs/criticisms/retaliation within the team	□ Yes	□ No
2.Ask what he/she/they can do to help the team succeed	□ Yes	□ No
3.Say something when/if I do something good for the team and/or team members	□ Yes	□ No
4. Thank me when/if I say there's a problem/issue with the team	□ Yes	□ No
5. Welcome input/feedback from the team	□ Yes	□ No
In the last month, the **athletic director** would have or did.?		
1.Check in about our team's progress/problems	□ Yes	□ No
2.Follow up with the coach/staff about things going well/poorly	□ Yes	□ No
3.Publicly celebrate the team's accomplishments with school leadership/media/sponsors	□ Yes	□ No
4. Thank me/my peers when/if we brought up an issue about the coach/staff	□ Yes	□ No
5.Welcome input/feedback from the team about the coach/staff	□ Yes	□ No
**Well-being**		
In the last week, my **coach** would have or did.?		
1.Check in with me about how I felt I was progressing	□ Yes	□ No
2.Say something when/if I do something good or better during training/exercises	□ Yes	□ No
3.Speak to me in a tone that conveyed respect	□ Yes	□ No
4. Acknowledge and make necessary adjustments for pain/injuries I have/had	□ Yes	□ No
5.Conduct any hands-on adjustments to my form or technique in a way that is useful and appropriate	□ Yes	□ No
In the last week, the **team doctor** would have or did.? □ n/a *(did not interact)*		
1.Take the time to check in with me about how I am feeling	□ Yes	□ No
2.Let me know that the doctor cares about me getting better	□ Yes	□ No
3.Speak to me in a tone that conveyed respect	□ Yes	□ No
4.Conduct any examinations in a way that is useful and appropriate	□ Yes	□ No

To assess well-being, athletes identify positive interactions, e.g., their coach speaking “in a tone that conveyed respect,” and “making necessary adjustments for pain/injury.” No questions directly ask about maltreatment. That said, reasonable inferences can certainly be drawn. If few to no positive interactions are indicated, athletes may be experiencing exploitation. If an abundance of positive interactions are identified, the team may be enjoying a near-absence of abuse and victimization—the ultimate goal.

To evaluate leaders' interactions, Komaki's reinforcement-based leadership model is used (Komaki, 1998). Athletes are asked whether coaches monitor (“checked in … about triumphs/criticisms/retaliation within the team”) and provide them with feedback (“say something when/if I do something good for the team”). Evidence for the model was provided, among other studies, by sailboat skippers during a round-robin regatta at the U.S. Naval Academy (Komaki et al., 1989). Winning skippers went beyond giving directives; they regularly inquired about their crews/sails as they shouted out words of encouragement.

To investigate team camaraderie, athletes indicate whether team members “celebrated with me when/if something good happens to me” and “intervened if someone bothered/pressured me.” As in the military, the social environment matters in sports. A close-knit culture can mitigate the often punishing conditions of training and competing. Ski powerhouse, the Norwegian men's Alpine team, exemplifies this idea: teammates share techniques, cheer for one another, work, play, and win together (Pennington, 2018; Cascagnette et al., 2020). Said Kjetil Jansrud, “if you have teammates who consistently lift you up, then … you'll work harder and stay motivated” (Pennington, 2018).

Athletes can be asked to complete the Checklist weekly on their phones (or other devices) so that input/feedback can be summarized on graphs. To ensure athletes are free of repercussion or retaliation, individual data are kept strictly confidential; only group scores are shared. No one is ever asked about wrongdoing. Instead, athletes indicate if their coach “welcomes input/feedback from the team.” Teams who have been retaliated against by coaches are less likely to indicate that their coaches embrace critiques.

Feedback graphs in hand, shout-outs can be given to coaches for responsible mentorship, to teammates for building camaraderie, and to athletic directors for broadcasting team accomplishments. Participants can judge progress, discuss suggestions for the following week, and adjust accordingly. Here, struggling coaches are given an opportunity to seek out guidance, while the athletic director can acknowledge coaches with strong improvement/accomplishments. Celebrations can be held monthly with results disseminated to top team officials and sponsors.

## Implications of the Proposed Paradigm Shift

This reinforcement model requires a paradigm shift: from wrongdoing to right-doing and from punishment to reward. Rather than ferreting out “bad apples,” the emphasis is on transforming the culture (Komaki and Minnich, 2016). Sports organizations of different levels could augment their current reporting/punishment process (U.S. Center for SafeSport, 2021a), as well as safety-risks-reduction programs (Kaufman et al., 2019; U.S. Center for SafeSport, 2021b) with a reinforcement initiative accentuating a positive culture. Furthermore, when hiring, coaches could be screened for evidence of supporting athlete well-being. Compensation packages could reward sports authorities for sustaining a positive culture.

Sports authorities have an opportunity here to follow the management adage “we treasure what we measure.” Rather than waiting for reports of abuse, Leung, for example, could use this safeguarding metric to proactively create “a safe, positive and encouraging environment where athlete voices are heard” (Allentuck, 2019). Using Checklist data, she could recognize coaches for right-doing and even brag to Sarah Hirshland, CEO of the U.S. Olympic and Paralympic Committee, about teams' safety and social health. In turn, Hirshland could highlight national governing body presidents who are successful in keeping athletes safe—possibly rewarding them with extra resources.

For coaches, receiving confidential, collective feedback about right-doing would open up opportunities for self-correction. Trying out new ideas might improve relationships with athletes and help ward off accusations of wrongdoing, evidently a grave fear of coaches (Tam et al., 2020). Recently barred from coaching for “severe aggressive behavior” (Macur, 2020b), Maggie Haney admitted making mistakes in the way in which she treated her athletes (Macur, 2020b,c). “Maybe what used to be OK is not OK anymore,” she said. Had Haney had real-time information about her athletes' responses to her coaching, she might have changed her interactions with them.

For athletes, a critical component of this paradigm shift is the empowerment—and subsequent prioritizing—of their voices. By using a safeguarding metric where athletes highlight positive behaviors (rather than being confined to reporting only negative behaviors), the power dynamic shifts. Furthermore, proactively building nurturing sports environments using positive consequences to motivate behavior would be an unorthodox but welcome change for athletes used to a “grin-and-bear-it,” “suck-it-up” environment (Pinches, 2020). Elite running coach Lauren Fleshman prides herself on checking in with athletes about their energy and mood, and making adjustments accordingly (Hamilton, 2020). Marathoner Carrie Mack said of Fleshman: “We state our own needs and they're accepted and heard… That's what's radical, and empowering.”

By galvanizing athletes and coaches around a positively embracing, athlete-centered culture, sports organizations can show they truly care, achieving Raisman's dream that no one would “ever ever have to say the words, ‘Me too”’ (Raisman, 2018).

## Author Contributions

All authors contributed to the article and approved the submitted version.

## Conflict of Interest

The authors declare that the research was conducted in the absence of any commercial or financial relationships that could be construed as a potential conflict of interest.

## Footnotes

^1^Here, we use “authority figures in sport” or “sports authorities” to refer broadly to people in sport settings with real or perceived power over athletes including coaches, athletic directors, club or team managers, physicians, national sport governing body presidents/directors, international federation presidents/directors, International Olympic or Paralympic Committee leadership, etc.
